# Supplementary material for: Image content is more important than Bouma’s Law for scene metamers
Source: eLife. 2019 Apr 30;8:e42512. doi: 10.7554/eLife.42512 (PMC6491040; doi:10.7554/eLife.42512)
Supplement: Transparent reporting form [file elife-42512-transrepform.docx]

***eLife’s* transparent reporting form**

We encourage authors to provide detailed information *within their submission* to facilitate the interpretation and replication of experiments. Authors can upload supporting documentation to indicate the use of appropriate reporting guidelines for health-related research (see [EQUATOR Network](http://www.equator-network.org/%20)), life science research (see the [BioSharing Information Resource](https://biosharing.org/)), or the [ARRIVE guidelines](http://www.plosbiology.org/article/info:doi/10.1371/journal.pbio.1000412) for reporting work involving animal research. Where applicable, authors should refer to any relevant reporting standards documents in this form.

If you have any questions, please consult our Journal Policies and/or contact us: [editorial@elifesciences.org](mailto:editorial@elifesciences.org).

**Sample-size estimation**

- You should state whether an appropriate sample size was computed when the study was being designed
- You should state the statistical method of sample size computation and any required assumptions
- If no explicit power analysis was used, you should describe how you decided what sample (replicate) size (number) to use

Please outline where this information can be found within the submission (e.g., sections or figure legends), or explain why this information doesn’t apply to your submission:

For most experiments in the paper we did not use an explicit power analysis. Rather, sample size was determined based on participant availability to achieve sample sizes typical of those in psychophysical studies. Performance for individual observers is in general very consistent, and we present both individual and population data in the manuscript to graphically demonstrate this consistency.

One of the supplementary experiments used an adaptive sample-size determination based on precision of model estimates. Details are provided in Appendix 2, Figure 5.

**Replicates**

- You should report how often each experiment was performed
- You should include a definition of biological versus technical replication
- The data obtained should be provided and sufficient information should be provided to indicate the number of independent biological and/or technical replicates
- If you encountered any outliers, you should describe how these were handled
- Criteria for exclusion/inclusion of data should be clearly stated
- High-throughput sequence data should be uploaded before submission, with a private link for reviewers provided (these are available from both GEO and ArrayExpress)

Please outline where this information can be found within the submission (e.g., sections or figure legends), or explain why this information doesn’t apply to your submission:

We have replicated the main result from the paper using new participants in a different psychophysical task (supplementary figure S5).

Criteria for inclusion / exclusion and treatment of outliers are stated throughout the paper. All criteria were determined prior to data collection but were not externally pre-registered.

**Statistical reporting**

- Statistical analysis methods should be described and justified
- Raw data should be presented in figures whenever informative to do so (typically when N per group is less than 10)
- For each experiment, you should identify the statistical tests used, exact values of N, definitions of center, methods of multiple test correction, and dispersion and precision measures (e.g., mean, median, SD, SEM, confidence intervals; and, for the major substantive results, a measure of effect size (e.g., Pearson's r, Cohen's d)
- Report exact p-values wherever possible alongside the summary statistics and 95% confidence intervals. These should be reported for all key questions and not only when the p-value is less than 0.05.

Please outline where this information can be found within the submission (e.g., sections or figure legends), or explain why this information doesn’t apply to your submission:

Statistical analysis is described in the Methods and in detail in the supplementary material. Code for analyses is provided. Data for individual participants is presented in figures.

Since we estimate the posterior distribution for mixed effect (multilevel) model parameters, commonly-reported statistics do not apply here (e.g. p-values). Furthermore, some values (e.g. effect sizes) are difficult to define for multilevel models. We have instead reported credible intervals on parameter estimates, the posterior probability for the parameter to be negative / positive, and model comparison based on information criteria as appropriate. We provide guidance on interpreting these values in the “Data Analysis” section of the Methods section.

(For large datasets, or papers with a very large number of statistical tests, you may upload a single table file with tests, Ns, etc., with reference to sections in the manuscript.)

**Group allocation**

- Indicate how samples were allocated into experimental groups (in the case of clinical studies, please specify allocation to treatment method); if randomization was used, please also state if restricted randomization was applied
- Indicate if masking was used during group allocation, data collection and/or data analysis

Please outline where this information can be found within the submission (e.g., sections or figure legends), or explain why this information doesn’t apply to your submission:

All experiments were conducted as within-subjects designs, meaning that each participant acts in all experimental conditions. No masking (double-blind control) was used at any stage of data collection or analysis.

**Additional data files (“source data”)**

- We encourage you to upload relevant additional data files, such as numerical data that are represented as a graph in a figure, or as a summary table
- Where provided, these should be in the most useful format, and they can be uploaded as “Source data” files linked to a main figure or table
- Include model definition files including the full list of parameters used
- Include code used for data analysis (e.g., R, MatLab)
- Avoid stating that data files are “available upon request”

Please indicate the figures or tables for which source data files have been provided:

All data, stimuli, analysis and stimulus-generating code is available (after publication) in a public repository at <http://dx.doi.org/10.5281/zenodo.1475112>

.
